# Minocycline attenuates interferon-α-induced impairments in rat fear extinction

**DOI:** 10.1186/s12974-016-0638-z

**Published:** 2016-06-30

**Authors:** Qiang Bi, Lijuan Shi, Pingting Yang, Jianing Wang, Ling Qin

**Affiliations:** Department of Physiology, China Medical University, Shenyang, 110001 People’s Republic of China; Department of Rheumatology and Immunology, First Affiliated Hospital, China Medical University, Shenyang, 110001 People’s Republic of China

**Keywords:** Fear conditioning, Extinction, Amygdala, Cytokine, Microglia, Astrocyte

## Abstract

**Background:**

Extinction of conditioned fear is an important brain function for animals to adapt to a new environment. Accumulating evidence suggests that innate immune cytokines are involved in the pathology of psychotic disorders. However, the involvement of cytokines in fear dysregulation remains less investigated. In the present study, we investigated how interferon (IFN)-α disrupts the extinction of conditioned fear and propose an approach to rescue IFN-α-induced neurologic impairment.

**Methods:**

We used a rat model of auditory fear conditioning to study the effect of IFN-α on the fear memory process. IFN-α was infused directly into the amygdala of rats and examined the rats’ behavioral response (freezing) to fear-conditioned stimuli. Immunohistochemical staining was used to examine the glia activity status of glia in the amygdala. The levels of the proinflammatory cytokines interleukin (IL)-1β and tumor necrosis factor (TNF)-α in the amygdala were measured by enzyme-linked immunosorbent assay. We also administrated minocycline, a microglial activation inhibitor, before the IFN-α infusion to testify the possibility to reverse the IFN-α-induced effects.

**Results:**

Infusing the amygdala with IFN-α impaired the extinction of conditioned fear in rats and activated microglia and astrocytes in the amygdala. Administering minocycline prevented IFN-α from impairing fear extinction. The immunohistochemical and biochemical results show that minocycline inhibited IFN-α-induced microglial activation and reduced IL-1β and TNF-α production.

**Conclusions:**

Our findings suggest that IFN-α disrupts the extinction of auditory fear by activating glia in the amygdala and provides direction for clinical studies of novel treatments to modulate the innate immune system in patients with psychotic disorders.

## Background

Extinction of fear is defined as a reduction in conditioned fear when a conditioned stimulus (CS) is repeatedly presented in the absence of an unconditioned stimulus (US). The inability to extinguish intense fear memories is an important clinical problem in patients with psychiatric disorders involving dysregulation of fear, such as specific phobias, panic disorder, and post-traumatic stress disorder [1–6]. Increasing interest has developed for the role of innate immune cytokines in impaired neuronal function and cognition that arise with trauma, infection, and/or disease. Several clinical studies have shown that levels of the innate immune cytokines, such as interleukin (IL)-1, IL-6, and tumor necrosis factor (TNF)-α, are correlated with impaired fear extinction [7–9]. However, the mechanism underlying the correlation between cytokines and psychiatric disorders remains unclear.

Behavioral tests designed to model this aspect of mental disorders are based on Pavlovian principles of associating an innocuous cue, such as a tone or light (CS), with a painful or aversive stimulus, such as an electrical foot-shock (US). Conditioned fear responses can then be indexed through various outputs, such as conditioned freezing [10]. Rodent models of Pavlovian conditioning have been widely used to study consolidation, extinction, and reconsolidation of fear memory [11]. Extinction in fear conditioning studies involves exposing rodents to a fear eliciting cue(s) or context without the aversive US [12, 13]. The fear extinction behavior is thought to be an active learning process and not simply “forgetting” a conditioned behavior that reverses the original learning [14].

It has been established that the basolateral amygdala (BLA) is a key brain structure for extinction learning [10]. To further reveal the pathological roles of cytokines in impaired fear memory, we directly tested whether microinfusion of interferon (IFN)-α into the amygdala affects auditory fear extinction in rats. IFN-α is an innate immune cytokine with antiviral and anti-proliferative activities and is therefore used to treat infectious diseases and cancers [15, 16]. The involvement of IFN-α in the brain function has been demonstrated by clinical studies showing that IFN-α induces high rates of behavioral disturbance, including depression, in 30–50 % of IFN-α-treated patients [10, 17–24]. Some experimental studies have explored the potential mechanisms of IFN-α-induced depression by systemic or intra-cerebroventricular injections of IFN-α in rodents [25–28]. However, no study has investigated the effects of IFN-α on fear extinction.

In this study, we first determined that directly infusing exogenous IFN-α into the amygdala impaired the extinction of conditioned fear in rats. Because glial cells play active roles in initiating and maintaining the inflammatory process in the brain, we further examined the activation of microglia and astrocytes in the amygdala following the IFN-α infusion. Next, we determined whether administering minocycline, an inhibitor of microglial activation, ameliorated IFN-α-induced impairment of fear extinction.

## Methods

### Subjects

This study conformed with the policies and procedures detailed in the “Guide for the Care and Use of Laboratory Animals” of the National Institutes of Health. The animal experimental protocols of the “Guide” and the treatment procedures were reviewed and approved by the Animal Care and Use Committee of China Medical University (No. 2014195). Male Wistar rats (weight, 250–300 g) from our own colony were housed in a humidity- (50–55 %) and temperature-controlled (22–24 °C) facility under a 12-h light/dark cycle (lights on at 7:30 a.m.). The animals had free access to food (standard laboratory rat chow) and water. All surgeries were performed under anesthesia, and all efforts were made to minimize animal suffering.

### Behavioral apparatus

The rats were fear conditioned in a 25 × 29 × 28-cm chamber (context A) constructed of aluminum and Plexiglas walls (Coulbourn Inst., Allentown, PA, USA). The floor consisted of stainless steel bars that could be electrified to deliver a mild shock, and illumination was provided by a single overhead light. The chamber had a speaker mounted on the outside wall and was placed inside a sound-attenuating box. The fear conditioning chambers were cleaned with 5 % ethanol each time a rat was removed from the chamber. Fear conditioning of the rats was extinguished and tested in context B, wherein the chamber was modified by introducing a black Plexiglas floor washed with peppermint soap. The wall pattern was changed to black and white stripes, and three house lights were installed. The CS was a 5-kHz tone with a 20-s duration and 75 dB intensity. The US was a 1.0-mA foot shock of 0.5-s duration, which co-terminated with the tone during the conditioning phase.

### Drugs

Recombinant human IFN-α was obtained from PeproTech Inc. (#300-02AB; Rocky Hill, NJ, USA) and was dissolved in artificial cerebrospinal fluid (ACSF; glucose, 5 mM; CaCl_2_, 1 mM; NaCl, 125 mM; MgCl_2_, 1 mM; NaHCO_3_, 27 mM; KCl, 0.5 mM; Na_2_SO_4_, 0.5 mM; NaH_2_PO_4_, 0.5 mM; and Na_2_HPO_4_, 1.2 mM). Rat serum albumin (1 mg) was added to 1 ml of 2 × 10^7^ IU/ml IFN-α. The rats received bilateral infusions of ACSF (vehicle) or IFN-α at doses of 100, 200, or 400 IU/μl (1 μl/side).

Minocycline hydrochloride (#M9511; Sigma, St. Louis, MO, USA) was dissolved fresh in 0.9 % NaCl and administered intragastrically (i.g.) once daily at a dosage of 90 mg/kg rat body weight for 3 days prior to the IFN-α treatment. The dose was selected on the basis of previous studies showing the beneficial effects of this dosage in animal models of cerebral brain ischemia, multiple sclerosis, and Parkinson’s disease [29–33].

### Cannula implantation and microinjections

The rats were anesthetized with sodium pentobarbital (50 mg/kg) intraperitoneally (i.p.) and mounted on a stereotaxic apparatus (SR-5R; Narishige, Tokyo, Japan) for surgery. Two cannulae consisting of a 22-gauge stainless steel tubing were implanted bilaterally into the BLA. The coordinates were AP, −2.3 mm; ML, ±4.5 mm; and DV, −7.0 mm according to the Paxinos and Watson brain atlas [34]. Three jewelry screws were implanted in the skull as anchors, and the entire assembly was affixed to the skull with dental cement. A 28-gauge dummy cannula was inserted into each cannula to prevent clogging. After the surgical procedure, the rats were monitored daily and given 1 week to recover prior to the experiment. The microinjections were performed at 10:00–12:00 a.m. IFN-α was dissolved in sterile ACSF and injected into the BLA via a 28-gauge infusion cannula connected with polyethylene (PE 20) tubing to a 10-μl Hamilton microsyringe (Hamilton Co., Reno, NV, USA). The infusion cannula protruded 0.5 mm beyond the guide cannula. An infusion volume of 1 μl was delivered using a Harvard PHD2000 syringe pump (Harvard Apparatus, Holliston, MA, USA) over the course of 10 min (at a rate of 0.1 μl/min). The infusion cannula remained in place for at least 1 min after the infusion before being pulled out to prevent backflow of the injectate through the guide cannula.

After all behavioral tests were completed, the rats were anesthetized with sodium pentobarbital (100 mg/kg, i.p.) and transcardially perfused with paraformaldehyde (4 %, pH 7.4). The brains were removed and sectioned, and slides were prepared and examined under a light microscope to verify that the cannulae were placed in the BLA. Only rats with proper placement of the cannulae were included in statistical analyses.

### Fear conditioning and extinction and drug application

Acclimation to the experimental conditions is an important measure to reduce unpredictable effects on behavior. Before the behavioral experiments, the rats were acclimated to handling and to the laboratory for 5 days. The rats were habituated to the test chamber for 30 min (contexts A and B for 15 min each in a counterbalanced manner) on day 5. The rats were placed in the context A chamber the next day and received a tone habituation session consisting of five CS presentations (5 kHz tone, 75 dB, 20 s). The rats’ behavior was measured as the baseline response. Next, the rats received seven tone–shock pairs in context A (conditioning session) for auditory fear conditioning.

We only selected rats for the extinction experiment that showed equivalent levels of behavioral response during the conditioning session to exclude the potential effects of the rat’s internal characteristics, surgery, or other health differences. The conditioned rats were divided into four groups. Each group received an infusion of either vehicle or IFN-α into the BLA (100, 200, or 400 IU/side, bilaterally) and was returned to their cage. The rats were placed in context B 8 h later and received an extinction session consisting of 15 tone–alone trials of 20 s each. The extinction session was designed to test the rat’s fear memory to the auditory CS rather than to the environment. Thus, we changed the wall pattern (blank to striped), floor material (steel to Plexiglas), illumination (one to three lights), and smell (ethanol to peppermint soap). The freezing behavior of all rats was scored by the same experimenter who was blind to the experimental conditions to reduce subjective error, and behavior was scored through a video camera. Freezing responses were judged as the absence of all movement except those related to respiration [35, 36]. The total duration of the freezing response during tone presentation (20 s) was recorded and transformed into a percentage of freezing (seconds spent freezing/20-s CS).

To evaluate whether minocycline could modulate the effects of IFN-α on extinction memory, the rats were assigned randomly to either the vehicle or minocycline group. Before the fear conditioning and extinction experiments, the rats received daily administration of the vehicle or minocycline at a dose of 90 mg/kg i.g. for 3 days. After the rats completed auditory fear conditioning in context A, they received an intra-amygdala infusion of IFN-α (400 IU/side). The rats underwent extinction training in context B 8 h later. Another group of rats received only minocycline without IFN-α to assess the effect of minocycline on fear extinction.

### Immunohistochemistry

Immediately following the behavioral experiments, the rats were deeply anesthetized with sodium pentobarbital (100 mg/kg, i.p.) and perfused via the ascending aorta with cold 0.9 % NaCl followed by chilled 4 % paraformaldehyde in 0.01 M phosphate-buffered saline (PBS). The brains were post-fixed in the same fixative for 24 h at 4 °C and embedded in paraffin for sectioning at 5 μm. Serial coronal sections were cut through the amygdala (at a level corresponding to 2–3 mm posterior to the bregma) [34].

Immunohistochemical staining was performed using the avidin–biotin–peroxidase complex detection kit and diaminobenzidine substrate. Microglial activation was measured using an antibody to ionized calcium-binding adaptor molecule 1 (Iba1; 1:100, goat polyclonal; Abcam, Shanghai, China). Astrocytic activation was measured using an antibody to glial fibrillary acidic protein (GFAP). Sections were incubated with their primary antibodies for 16 h at 4 °C. Negative control sections were incubated with PBS instead of primary antibodies. The sections were incubated with the appropriate avidin–biotin complex solutions (Zhongshan Golden Bridge, Beijing, China) at 37 °C for 20 min. All sections were counterstained with Harris’s hematoxylin.

### Cell quantification

To minimize any potential confounding effects from immunohistochemistry, the sections were prepared, stained, and imaged at the same time as their relevant control. Furthermore, the cell number was counted in a predefined area of the brain. Nine sections among the serial coronal sections of the amygdala were selected from each brain, which were centered at the site of the cannula tip and separated by 10 sections (50 μm). The areas of the amygdala were captured using an Olympus BX51 automatic microscope (Tokyo, Japan). The total numbers of cells stained with GFAP, Iba1, or neuronal nuclear antigen (NeuN) in a 400 × 400 μm area (cannula tip centered) were marked by an operator who was blinded to the identity of the sections, and an automated cell count was generated using an image analysis system. Only morphologically intact and clearly identifiable cells were counted in the regions. As no obvious difference in cell profiles was detected between the two hemispheres, the right and left hemisphere values were averaged for each rat. The number of cells in each section was averaged to obtain a mean value for each animal (nine sections/rat). The mean values obtained from five rats in each group were used for the statistical analysis.

### Measurement of proinflammatory cytokines

IL-1β and TNF-α levels in the amygdala were measured using enzyme-linked immunosorbent assay (ELISA) kits according to the manufacturer’s instructions (Neobioscience, Shenzhen, China). The rats were deeply anesthetized with sodium pentobarbital (100 mg/kg, i.p.), and the brain was removed rapidly and frozen at −20 °C for 20 min. To reduce the possible error caused by brain sampling, we placed the frozen brain on a rat brain section mold (# 68709; RWD Life Science, Shenzhen, China) and carefully dissected the brain tissues corresponding to the amygdala (AP −1.0 to −4.0 mm, ML 4.0–6.0 mm, and DV 7.0–9.0 mm) using a sharp steel blade. Brain tissues from both hemispheres were mixed and homogenized on ice in 0.01 M PBS (pH, 7.4) and centrifuged at 12,000 rpm for 15 min at 4 °C. The supernatants were collected and stored at −80 °C until the measurement of IL-1β and TNF-α by ELISA. All samples were measured in duplicate and adjusted according to the protein content determined using an enhanced BCA Protein Assay kit (Beyotime, Harman, China). The results are expressed as picogram per milligram protein.

### Statistical analyses

Statistical analyses were performed using SPSS 18.0 software (SPSS Inc., Chicago, IL, USA). Depending on whether data were normally distributed or not (determined using the Kolmogorov–Smirnov test), either parametric or nonparametric test was used for statistical evaluation. Two-way repeated-measures analysis of variance (ANOVA) was performed on the freezing response data among the different groups and trials. Differences in the immunohistochemical data and ELISA results were detected by one-way ANOVA. Each ANOVA reporting significant effects was followed by Tukey’s post hoc test of multiple comparison. A *p* value of <0.05 was considered statistically significant.

## Results

### Intra-amygdala infusion of IFN-α impairs extinction of conditioned fear in a dose-dependent manner

**Fig. 1:**
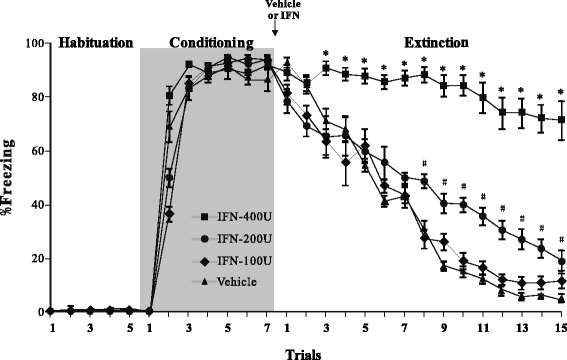
Effect of interferon (IFN)-α treatment on fear extinction. Mean ± standard error of percent freezing compared to that of the vehicle. Rats (*n* = 8/group) were treated with 100, 200, or 400 IU IFN-α for the habituation, conditioning, and extinction trials. Vehicle or IFN-α was administrated after the rats experienced the habituation and conditioning trials. *p < 0.05 in the comparison (Tukey’s test) between vehicle and 400 IU IFN-α group. ^#^ *p* < 0.05 in the comparison between vehicle and 200 IU IFN-α group

The effect of IFN-α on the extinction of conditioned fear was assessed by data from 32 rats. The rats were assigned into four groups of eight animals each matched for their freezing response during auditory fear conditioning. After fear conditioning in context A, they received one of the following treatments: ACSF (vehicle group), 100 IU IFN-α (low-dose group), 200 IU IFN-α (medium-dose group), or 400 IU IFN-α (high-dose group). The rats were tested for fear extinction 8 h later in context B. Figure 1 shows the mean and standard error of the freezing responses in the rat groups during the habituation, conditioning, and extinction sessions. Rats in all groups displayed a low freezing level during the habituation session, indicating normal locomotor activity. The rats showed a high freezing level after experiencing two CS (5 kHz tone) and US (foot shock) trials during the conditioning session, suggesting that they quickly learned the association between the CS and the US. No difference in the freezing percentage was observed among the groups during the habituation and conditioning sessions. However, the freezing percentage in the vehicle group diminished gradually with repeated presentation of the CS alone (two-way ANOVA for trial, *F*_14,420_ = 74.2, *p* < 0.001) and administration of IFN-α significantly reduced extinction (two-way ANOVA for group, *F*_3,420_ = 66.4, *p* < 0.001). The interaction between the experimental groups and trials was significant (two-way ANOVA for interaction, *F*_42,420_ = 6.2, *p* < 0.001), indicating that the behavioral effects of the different treatments may have varied inconsistently by trial. Therefore, we performed Tukey’s multiple comparison tests to clarify the differences between each pair of groups in each trial. A significant effect was observed after correcting for multiple testing during trials 3–15 when the rats received 400 IU IFN-α, and during trials 8–15 when rats received 200 IU IFN-α (*p* < 0.05 for pairwise comparisons between vehicle vs. 400 IU and vehicle vs. 200 IU; indicated by asterisk and pound sign in Fig. 1). No difference was observed between the vehicle and 100 IU IFN-α groups throughout the extinction session. These results indicate a dose-dependent inhibitory effect of IFN-α on extinction. Because 400 IU IFN-α produced the maximal impairment on extinction, we used this dose in subsequent experiments.

### Activation of microglia and astrocytes following IFN-α infusion

**Fig. 2:**
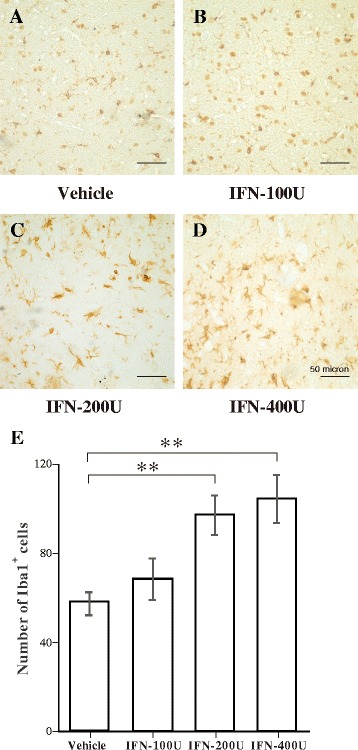
Immunohistochemical analysis of Iba1-immunopositive microglia in the amygdala following vehicle or interferon (IFN)-α administration. **a**–**d** Representative microphotographs showing changes in the number of Iba1-immunopositive cells in the amygdala after vehicle, 100, 200, or 400 IU IFN-α was administered. **e** Quantitative analysis of the number of Iba1-immunopositive microglia in predefined areas of the amygdala. **p < 0.01, analysis of variance followed by Tukey’s test; *n* = 5 animals/group

The activation status of microglia and astrocytes was examined in the amygdala of rats following the behavioral experiments. Resting microglia in the vehicle group exhibited low Iba1-immunoreactivity and had small cell bodies with extensive ramifications in the amygdala (Fig. 2a). Iba1-immunoreactivity increased in the 100 and 200 IU IFN-α groups, indicating the activation of microglia (Fig. 2b, c). The strongest Iba1-immunoreactivity was found in the 400 IU IFN-α group, in which microglia had large cell bodies with short processes, and some of them showed a phagocytic shape (Fig. 2d). Quantitative analysis by one-way ANOVA revealed that the number of Iba1-immunopositive microglia in the amygdala increased significantly in the 200 and 400 IU IFN-α groups compared with that in the vehicle group (Fig. 2e).

**Fig. 3:**
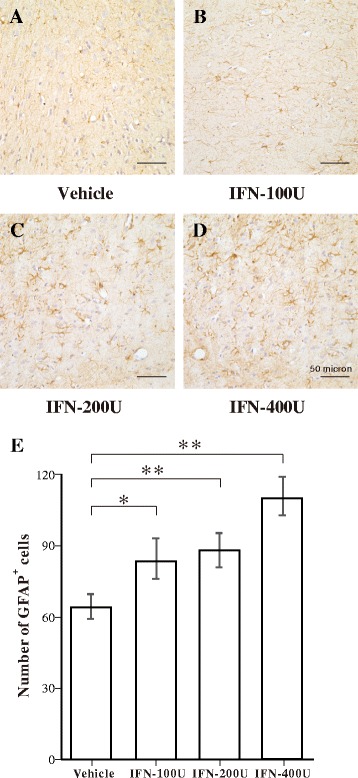
Immunohistochemical analysis of green fluorescent protein (GFAP)-immunopositive astrocytes in the amygdala following vehicle or interferon (IFN)-α administration. **a**–**d** Representative microphotographs showing changes in the number of GFAP-immunopositive cells in the amygdala after vehicle, 100, 200, or 400 IU IFN-α was administered. **e** Quantitative analysis of the number of GFAP-immunopositive astrocytes in predefined areas of the amygdala. **p < 0.01; *p < 0.05, analysis of variance followed by Tukey’s test; *n* = 5 animals/group

Figure 3 shows the status of astrocytes. Resting astrocytes in the vehicle group showed low GFAP-immunoreactivity and stellate-shaped cell bodies with long and thin processes in the amygdala (Fig. 3a). Astrocytes in the 100 and 200 IU IFN-α groups were activated, as indicated by increased GFAP-immunoreactivity and cell body (Fig. 3b, c). Activated astrocytes in the 400 IU IFN-α group were highly immunoreactive to GFAP and had a hypertrophic morphology, characterized by large cell bodies and thick processes (Fig. 3d). The quantitative analysis by one-way ANOVA revealed a significant increase in the number of GFAP-immunopositive astrocytes in the 100, 200, and 400 IU IFN-α groups (Fig. 3e).

**Fig. 4:**
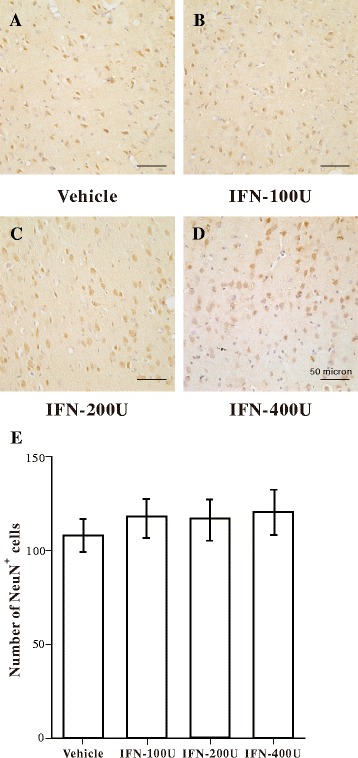
Immunohistochemical analysis of neuronal nuclear antigen (NeuN)-immunopositive neurons in the amygdala following vehicle or interferon (IFN)-α administration. **a**–**d** Representative microphotographs showing changes in the number of NeuN-immunopositive cells in the amygdala following vehicle, 100, 200, or 400 IU IFN-α administration. **e** Quantitative analysis of the number of NeuN-immunopositive neurons in the amygdala

We also used NeuN-immunostained sections to evaluate whether the IFN-α infusion induced neuronal loss in the amygdala. As shown in Fig. 4a–e, no difference in NeuN immunoreactivity was detected among the groups. Thus, the infusion of IFN-α did not cause obvious neuronal loss in the amygdala.

### Minocycline attenuates IFN-α-induced impairment of extinction

**Fig. 5:**
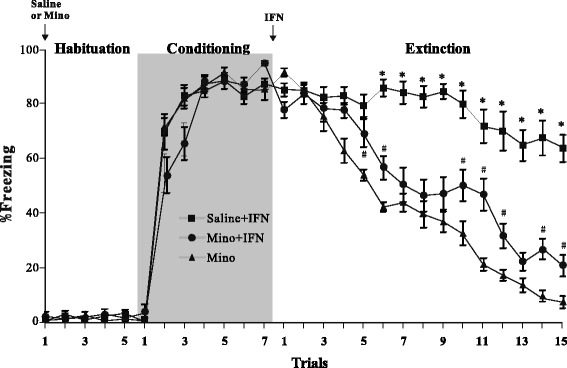
Effect of minocycline on interferon (IFN)-α-induced impairment of fear extinction. Mean ± standard error of percent freezing to tone in minocycline, saline + IFN-α, and minocycline + IFN-α-treated rats (*n* = 10/group) across the habituation, conditioning, extinction, and trials. Saline or minocycline was administered before the behavior experiment. IFN-α was administered after the conditioning trials. *p < 0.05 in the comparison (Tukey’s test) between saline + IFN-α and minocycline + IFN-α group. ^#^ *p* < 0.05 in the comparison between minocycline and minocycline + IFN-α group

Because the in vitro experiment indicated that the IFN-α infusion activated glia, we further examined whether minocycline, an inhibitor of microglial activation, reduced the behavioral impairments induced by IFN-α-treatment. One group of rats (*n* = 10) received an i.g. administration of minocycline (90 mg/kg, dissolved in saline) for 3 days before the auditory fear conditioning and infusion of 400 IU IFN-α (minocycline + IFN-α group). The second group (*n* = 10) received the same dose of saline and IFN-α as the control group (saline + IFN-α group). The third group (*n* = 10) received only minocycline without the IFN-α infusion (minocycline group). All rats participated in the fear extinction experiment 24 h after treatment with minocycline or saline. Figure 5 shows the percentage of time that the rats spent freezing during the habituation, conditioning, and extinction sessions. Rats pretreated with minocycline and saline showed similar freezing response levels during the habituation and conditioning sessions, suggesting that minocycline had no effect on locomotor activity or acquisition of fear conditioning. Rats in the minocycline group showed a gradual decrease of freezing time during the extinction session, indicating that fear extinction was not disrupted by a single application of minocycline. Rats in the saline + IFN-α group showed delayed extinction of the CS-associated freezing response, similar to the results observed in rats receiving only the 400 IU IFN-α infusion (Fig. 1). As expected, combined application of minocycline and IFN-α rescued the IFN-α induced deficit in fear conditioning extinction. Two-way ANOVA revealed a significant main effect of the treatment group (*F*_2,405_ = 36.3, *p* < 0.001) and trial (*F*_14,405_ = 52.0, *p* < 0.001). A significant interaction was observed between treatment and trial (*F*_28,405_ = 6.9, *p* < 0.001), indicating dependency of the treatment effect on the trial. Tukey’s multiple comparison tests further revealed that the freezing percentage in the minocycline + IFN-α group was significantly lower than that in the saline + IFN-α group during extinction session trials 6 and 15 (*p* < 0.05, asterisk in Fig. 5), suggesting that minocycline attenuated the IFN-α-induced impairment of fear extinction. The freezing percentage in the minocycline + IFN-α group remained higher than that in the minocycline group in some trials (*p* < 0.05). Thus, minocycline alone did not completely restore fear-extinction capability.

### Minocycline inhibits IFN-α-induced microglial activation without interfering with reactive astrocytes

**Fig. 6:**
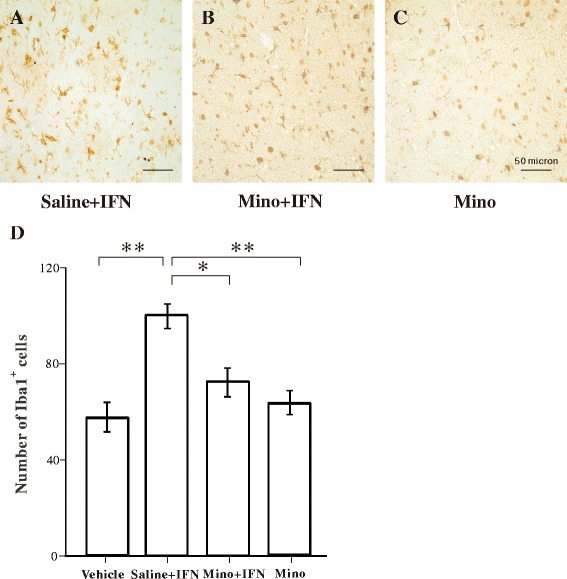
Effect of minocycline on interferon (IFN)-α-induced microglial activation in the amygdala. **a**–**c** Representative microphotographs showing changes in Iba1-immunopositive microglia in the amygdala in the saline + INF-α, minocycline + INF-α, and minocycline groups. **d** Quantitative analysis of the number of Iba1-immunopositive microglia. **p < 0.01; *p < 0.05, analysis of variance followed by Tukey’s test; *n* = 5 animals/group

We further examined the effects of minocycline on IFN-α-induced glial activation in the amygdala. Microglia were clearly activated in the saline + IFN-α group (Fig. 6a), compared with the results of the vehicle group shown in Fig. 3a. The combination of minocycline and IFN-α reduced the extent of microglial activation (Fig. 6b), and a single application of minocycline did not activate microglia (Fig. 6c). Consistent with these observations, one-way ANOVA and Tukey’s post hoc test revealed that the number of Iba1-immunopositive cells in the saline + IFN-α group was significantly higher than those in the other groups (Figs. 6d). No significant difference in the number of Iba1-immunopositive cells was detected between the vehicle and minocycline groups, indicating that minocycline inhibited IFN-α induced activation of microglia but did not affect microglia under physiological conditions.

**Fig. 7:**
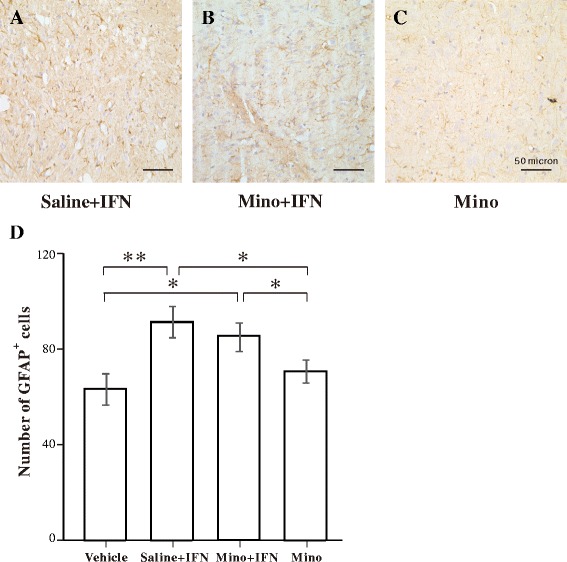
Effect of minocycline on interferon (IFN)-α-induced astrocytic activation in the amygdala. **a**–**c** Representative microphotographs showing changes in green fluorescent protein (GFAP)-immunopositive astrocytes in the amygdala in the saline + INF-α, minocycline + INF-α, and minocycline groups. **d** Quantitative analysis of the number of GFAP-immunopositive astrocytes. **p < 0.01; *p < 0.05, analysis of variance followed by Tukey’s test; *n* = 5 animals/group

In contrast, the number of GFAP-immunoreactive astrocytes was not significantly different between the saline + IFN-α and the minocycline + IFN-α groups (Fig. 7), but the numbers in both groups were significantly higher than those in the vehicle and minocycline groups. These results suggest that minocycline did not inhibit IFN-α-induced astrocytic activation.

### Minocycline inhibits IFN-α-induced IL-1β and TNF-α production

**Fig. 8:**
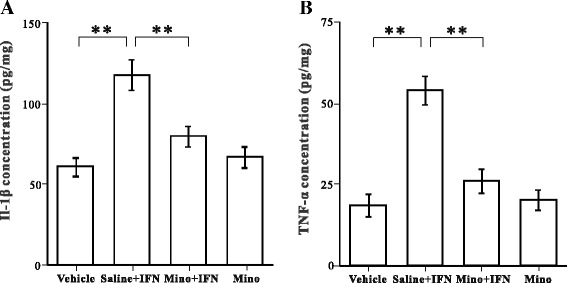
Effect of minocycline on interleukin (IL)-1β and tumor necrosis factor (TNF)-α concentrations in the amygdala. *Bar graphs* illustrate the concentrations of IL-1β (**a**) and TNF-α (**b**) in the amygdala in the vehicle, saline + INF-α, minocycline +I NF-α, and minocycline groups. **p < 0.01, analysis of variance followed by Tukey’s test; *n* = 5 animals/group

We used ELISA to evaluate the effects of minocycline on proinflammatory cytokine (IL-1β and TNF-α) production in the vehicle, saline + IFN-α, minocycline + IFN-α, and minocycline groups. The results (Fig. 8) show that IL-1β and TNF-α concentrations in the amygdala increased significantly in the saline + IFN-α group, compared to those in the vehicle group, whereas treatment with minocycline + IFN-α significantly decreased the IFN-α-induced increase in IL-1β and TNF-α concentrations (one-way ANOVA and Tukey’s post hoc test, *p* < 0.001). IL-1β and TNF-α concentrations were similar in the vehicle and minocycline groups, demonstrating that minocycline did not affect the normal cytokine levels in the brain.

## Discussion

In the present study, to the best of our knowledge, we demonstrated for the first time that intra-amygdala infusion of IFN-α impaired the extinction of conditioned fear in a dose-dependent manner. This result is consistent with previous findings that intra-amygdala infusion of IL-6 and TNF-α interrupts auditory fear conditioning behavior in rats [36, 37]. We further found that the detrimental effect of IFN-α was prevented by pretreatment with the microglial activation inhibitor minocycline. In addition, the immunohistochemical and biochemical results demonstrate that minocycline inhibited IFN-α-induced microglial activation and increased the production of IL-1β and TNF-α. These findings highlight the role of glial activation in mental disorders associated with innate immunity and underpin the disease-modifying activity of minocycline.

### Glial activation induced by IFN-α

IFN-α is an innate immune cytokine with anti-viral, anti-proliferative, and apoptotic effects, as well as boosting the immune system. IFN-α is often used to treat hepatitis-C and some cancers (hematological malignancies, leukemia and lymphomas, and melanoma) [38–40]. However, long-term IFN-α treatment frequently triggers a variety of neuropsychiatric symptoms, such as depression [28, 40–44]. Zheng et al. reported that systemic IFN-α administration activates microglia in the hippocampus, which may mediate the development of IFN-α-induced depression [28]. This finding is in accordance with our result showing that IFN-α activated microglia in the amygdala. Microglia are the resident immune cells in the brain and are integral to inflammatory processes during central nervous system (CNS) disease states [45]. Activation of microglia is associated with increased release of IL-1β and TNF-α, both of which promote hyperexcitability and hypersynchrony via transcriptional or post-transcriptional mechanisms [33, 46]. Our results also show that the levels of IL-1β and TNF-α increased in response to the IFN-α infusion in the amygdala. A study of depression showed that IFN-α treatment induces secretion of proinflammatory cytokines from microglia, which suppress neurogenesis in the hippocampus [28]. Thus, the authors suggested that decreased hippocampal neurogenesis plays an important role in the development of depression. The main difference between our present study and previous studies on depression is the method of IFN-α administration. We directly infused a single dose of IFN-α into the amygdala, whereas previous studies adopted 4–5 weeks of systemic IFN-α administration. In the present study, we found no clear change in the number or morphology of neurons in the amygdala, suggesting that the observed impairment in fear extinction was not caused by a structural change in the neural circuit. Our behavioral and histological measurements may have been conducted too early (8 h post IFN-α administration) to observe a clear neuropathological change. Nevertheless, our results suggest that the fear memory function can be disrupted at the early phase of the inflammatory response in the brain.

Our results also show that astrocytes were activated by infusing IFN-α into the amygdala. Astrocytes are the major glial cell population in the CNS and are active participants in propagating and regulating neuroinflammation [47–50]. Two potential mechanisms of astrocyte-mediated brain impairment have been proposed. First, studies using astrocyte cultures have revealed that astrocytes secrete cytokines in response to stimulation by inflammatory cytokines [51–53]. Thus, astrocytes activated by IFN-α in the amygdala may be a source of overexpressed IL-1β and TNF-α, which could disturb normal neuronal activities [44, 54]. Second, astrocytes regulate the synaptic transmission of neurons via uptake or release of neurotransmitters [55]. For example, reactive astrocytes produce the inhibitory gliotransmitter gamma aminobutyric acid, which reduces synaptic plasticity and the performance of learning and memory in a mouse model [56]. The second mechanism may be more appropriate for our present results (see below).

### Protective effects of minocycline

Another important finding in our study was that minocycline prevented IFN-α-induced impairment of fear extinction. Minocycline is a tetracycline derivative that has powerful anti-inflammatory, anti-apoptotic, and antioxidant properties independent of its antibacterial activity. Because of its high lipid solubility, peripherally administrated minocycline easily crosses the blood–brain barrier [28, 57]. The neuroprotective effect of minocycline may be partially mediated by its ability to inhibit IFN-α-induced microglial activation and concomitant production of proinflammatory cytokines, as demonstrated in Figs. 6 and 8. Compelling evidence from in vivo and in vitro models of neurological disease demonstrates that minocycline exerts neuroprotective effects [28, 33, 58, 59].

In the present study, we found that astrocytes remained reactive after pretreatment with minocycline, as demonstrated by GFAP immunostaining in Fig. 7. Thus, minocycline did not inhibit astrocyte activation, which may explain why minocycline did not completely rescue IFN-α-induced behavioral impairment. The finding that minocycline prevented microglial activation without affecting reactive astrocytes is interesting, as astrocyte activation is a prominent feature in the neuroinflammed brain [60]. One possible explanation is that IFN-α-induced activation of microglia and astrocytes is mediated through different pathways, which is in line with previous studies showing distinct regulation of microglial and astrocyte activation after spreading depression and lipopolysaccharide-induced hyperalgesia [50, 61, 62].

Although astrocytes remained activated after minocycline was administered, IL-1β and TNF-α concentrations in the examined brain area had returned to normal levels (Fig. 8). Therefore, minocycline may prevent the increase in inflammatory cytokines in astrocytes without affecting their general activation state. Several in vivo studies have demonstrated that astrocytes remain morphologically activated in brain tissue after cytokine synthesis is inhibited [29, 63]. Because astrocytes are intimately involved in various functions, such as ion buffering, regulation of neurotransmission, and modulation of blood–brain barrier permeability in the normal brain [64, 65], activation of astrocytes may not only be associated with mediating the inflammatory responses in these cells. Furthermore, although astrocytes can produce proinflammatory cytokines like microglia, activated astrocytes may serve as immune effector cells that limit the immune response by producing anti-inflammatory cytokines [66]. For instance, astrocytes in in vivo seizure models are a key source of anti-inflammatory molecules, such as the IL-1 receptor antagonist [67], an endogenous competitive IL-1 receptor blocker, which impairs electrical kindling development in rats [68]. However, the potential roles of the various glial functions in neuroinflammatory response remain poorly understood and will likely be further investigated [33].

## Conclusions

The results of our study implicate a microglial-mediated mechanism in the development of IFN-induced impairments of fear extinction. Further studies are warranted to determine the precise mechanism by which minocycline improves fear extinction.

## Abbreviations

ACSF, artificial cerebrospinal fluid; ANOVA, analysis of variance; BLA, basolateral amygdala; CS, conditioned stimulus; ELISA, enzyme-linked immunosorbent assay; GFAP, glial fibrillary acidic protein; Iba1, calcium-binding adaptor molecule 1; IFN, interferon; IL-1β, interleukin-1β; IL-6, interleukin-6; NeuN, neuron-specific nuclear protein; PBS, phosphate-buffered saline; TNF-α, tumor necrosis factor-α; US, unconditioned stimulus

## References

[1] Gill J, Vythilingam M, Page GG (2008). Low cortisol, high DHEA, and high levels of stimulated TNF-alpha, and IL-6 in women with PTSD. J Trauma Stress.

[2] Gorman JM, Kent JM, Sullivan GM, Coplan JD (2000). Neuroanatomical hypothesis of panic disorder, revised. Am J Psychiatry.

[3] Guo M, Liu T, Guo JC, Jiang XL, Chen F, Gao YS (2012). Study on serum cytokine levels in posttraumatic stress disorder patients. Asian Pac J Trop Med.

[4] Fyer AJ (1998). Current approaches to etiology and pathophysiology of specific phobia. Biol Psychiatry.

[5] Lindqvist D, Wolkowitz OM, Mellon S, Yehuda R, Flory JD, Henn-Haase C, Bierer LM, Abu-Amara D, Coy M, Neylan TC (2014). Proinflammatory milieu in combat-related PTSD is independent of depression and early life stress. Brain Behav Immun.

[6] Rohleder N, Joksimovic L, Wolf JM, Kirschbaum C (2004). Hypocortisolism and increased glucocorticoid sensitivity of pro-inflammatory cytokine production in Bosnian war refugees with posttraumatic stress disorder. Biol Psychiatry.

[7] Andrews JA, Neises KD (2012). Cells, biomarkers, and post-traumatic stress disorder: evidence for peripheral involvement in a central disease. J Neurochem.

[8] Quinones MM, Maldonado L, Velazquez B, Porter JT (2016). Candesartan ameliorates impaired fear extinction induced by innate immune activation. Brain Behav Immun.

[9] Gola H, Engler H, Sommershof A, Adenauer H, Kolassa S, Schedlowski M, Groettrup M, Elbert T, Kolassa IT (2013). Posttraumatic stress disorder is associated with an enhanced spontaneous production of pro-inflammatory cytokines by peripheral blood mononuclear cells. BMC Psychiatry.

[10] Fendt M, Fanselow MS (1999). The neuroanatomical and neurochemical basis of conditioned fear. Neurosci Biobehav Rev.

[11] Shin LM, Liberzon I (2010). The neurocircuitry of fear, stress, and anxiety disorders. Neuropsychopharmacology.

[12] Quirk GJ, Garcia R, Gonzalez-Lima F (2006). Prefrontal mechanisms in extinction of conditioned fear. Biol Psychiatry.

[13] Peters J, Kalivas PW, Quirk GJ (2009). Extinction circuits for fear and addiction overlap in prefrontal cortex. Learn Mem.

[14] Bouton ME (2004). Context and behavioral processes in extinction. Learn Mem.

[15] Kirkwood J (2002). Cancer immunotherapy: the interferon-alpha experience. Semin Oncol.

[16] Dorr RT (1993). Interferon-alpha in malignant and viral diseases. A review. Drugs.

[17] Musselman DL, Lawson DH, Gumnick JF, Manatunga AK, Penna S, Goodkin RS, Greiner K, Nemeroff CB, Miller AH (2001). Paroxetine for the prevention of depression induced by high-dose interferon alfa. N Engl J Med.

[18] Raison CL, Demetrashvili M, Capuron L, Miller AH (2005). Neuropsychiatric adverse effects of interferon-alpha: recognition and management. CNS Drugs.

[19] Lotrich FE, Rabinovitz M, Gironda P, Pollock BG (2007). Depression following pegylated interferon-alpha: characteristics and vulnerability. J Psychosom Res.

[20] Asnis GM, De La Garza R (2006). Interferon-induced depression in chronic hepatitis C: a review of its prevalence, risk factors, biology, and treatment approaches. J Clin Gastroenterol.

[21] Orru MG, Baita A, Sitzia R, Costa A, Muntoni E, Landau S, Chessa L, Farci MG, Carpiniello B, Pariante CM (2005). [Interferon-alpha-induced psychiatric side effects in patients with chronic viral hepatitis: a prospective, observational, controlled study]. Epidemiol Psichiatr Soc.

[22] Bonaccorso S, Marino V, Puzella A, Pasquini M, Biondi M, Artini M, Almerighi C, Verkerk R, Meltzer H, Maes M (2002). Increased depressive ratings in patients with hepatitis C receiving interferon-alpha-based immunotherapy are related to interferon-alpha-induced changes in the serotonergic system. J Clin Psychopharmacol.

[23] Smith KJ, Norris S, O’Farrelly C, O’Mara SM (2011). Risk factors for the development of depression in patients with hepatitis C taking interferon-alpha. Neuropsychiatr Dis Treat.

[24] Maes M, Bonaccorso S (2004). Lower activities of serum peptidases predict higher depressive and anxiety levels following interferon-alpha-based immunotherapy in patients with hepatitis C. Acta Psychiatr Scand.

[25] Wang J, Dunn AJ, Roberts AJ, Zhang H (2009). Decreased immobility in swimming test by homologous interferon-alpha in mice accompanied with increased cerebral tryptophan level and serotonin turnover. Neurosci Lett.

[26] Minc JI (1949). Neuro-somato-vegetative disorders during early periods of closed war-time cranial injuries. Nevropatol Psikhiatriia.

[27] Lu DY, Leung YM, Su KP (2013). Interferon-alpha induces nitric oxide synthase expression and haem oxygenase-1 down-regulation in microglia: implications of cellular mechanism of IFN-alpha-induced depression. Int J Neuropsychopharmacol.

[28] Zheng LS, Kaneko N, Sawamoto K (2015). Minocycline treatment ameliorates interferon-alpha- induced neurogenic defects and depression-like behaviors in mice. Front Cell Neurosci.

[29] Yrjanheikki J, Keinanen R, Pellikka M, Hokfelt T, Koistinaho J (1998). Tetracyclines inhibit microglial activation and are neuroprotective in global brain ischemia. Proc Natl Acad Sci U S A.

[30] Yrjanheikki J, Tikka T, Keinanen R, Goldsteins G, Chan PH, Koistinaho J (1999). A tetracycline derivative, minocycline, reduces inflammation and protects against focal cerebral ischemia with a wide therapeutic window. Proc Natl Acad Sci U S A.

[31] Popovic N, Schubart A, Goetz BD, Zhang SC, Linington C, Duncan ID (2002). Inhibition of autoimmune encephalomyelitis by a tetracycline. Ann Neurol.

[32] Wu DC, Jackson-Lewis V, Vila M, Tieu K, Teismann P, Vadseth C, Choi DK, Ischiropoulos H, Przedborski S (2002). Blockade of microglial activation is neuroprotective in the 1-methyl-4-phenyl-1,2,3,6-tetrahydropyridine mouse model of Parkinson disease. J Neurosci.

[33] Wang N, Mi X, Gao B, Gu J, Wang W, Zhang Y, Wang X (2015). Minocycline inhibits brain inflammation and attenuates spontaneous recurrent seizures following pilocarpine-induced status epilepticus. Neuroscience.

[34] Paxinos G, Watson C (1997). The rat brain in stereotaxic coordinates.

[35] Blanchard RJ, Blanchard DC (1969). Passive and active reactions to fear-eliciting stimuli. J Comp Physiol Psychol.

[36] Jing H, Hao Y, Bi Q, Zhang J, Yang P (2015). Intra-amygdala microinjection of TNF-alpha impairs the auditory fear conditioning of rats via glutamate toxicity. Neurosci Res.

[37] Hao Y, Jing H, Bi Q, Zhang J, Qin L, Yang P (2014). Intra-amygdala microinfusion of IL-6 impairs the auditory fear conditioning of rats via JAK/STAT activation. Behav Brain Res.

[38] Deutsch M, Hadziyannis SJ (2008). Old and emerging therapies in chronic hepatitis C: an update. J Viral Hepat.

[39] Tagliaferri P, Caraglia M, Budillon A, Marra M, Vitale G, Viscomi C, Masciari S, Tassone P, Abbruzzese A, Venuta S (2005). New pharmacokinetic and pharmacodynamic tools for interferon-alpha (IFN-alpha) treatment of human cancer. Cancer Immunol Immunother.

[40] Zheng LS, Hitoshi S, Kaneko N, Takao K, Miyakawa T, Tanaka Y, Xia H, Kalinke U, Kudo K, Kanba S (2014). Mechanisms for interferon-alpha-induced depression and neural stem cell dysfunction. Stem Cell Reports.

[41] Dieperink E, Willenbring M, Ho SB (2000). Neuropsychiatric symptoms associated with hepatitis C and interferon alpha: a review. Am J Psychiatry.

[42] Bonaccorso S, Puzella A, Marino V, Pasquini M, Biondi M, Artini M, Almerighi C, Levrero M, Egyed B, Bosmans E (2001). Immunotherapy with interferon-alpha in patients affected by chronic hepatitis C induces an intercorrelated stimulation of the cytokine network and an increase in depressive and anxiety symptoms. Psychiatry Res.

[43] Lieb K, Engelbrecht MA, Gut O, Fiebich BL, Bauer J, Janssen G, Schaefer M (2006). Cognitive impairment in patients with chronic hepatitis treated with interferon alpha (IFNalpha): results from a prospective study. Eur Psychiatry.

[44] Hoyo-Becerra C, Schlaak JF, Hermann DM (2014). Insights from interferon-alpha-related depression for the pathogenesis of depression associated with inflammation. Brain Behav Immun.

[45] Gomes-Leal W (2012). Microglial physiopathology: how to explain the dual role of microglia after acute neural disorders?. Brain Behav.

[46] Vezzani A, Friedman A, Dingledine RJ (2013). The role of inflammation in epileptogenesis. Neuropharmacology.

[47] Farina C, Aloisi F, Meinl E (2007). Astrocytes are active players in cerebral innate immunity. Trends Immunol.

[48] Liu S, Kielian T (2011). MyD88 is pivotal for immune recognition of Citrobacter koseri and astrocyte activation during CNS infection. J Neuroinflammation.

[49] Norden DM, Fenn AM, Dugan A, Godbout JP (2014). TGFbeta produced by IL-10 redirected astrocytes attenuates microglial activation. Glia.

[50] Norden DM, Trojanowski PJ, Villanueva E, Navarro E, Godbout JP (2016). Sequential activation of microglia and astrocyte cytokine expression precedes increased iba-1 or GFAP immunoreactivity following systemic immune challenge. Glia.

[51] Cartier L, Hartley O, Dubois-Dauphin M, Krause KH (2005). Chemokine receptors in the central nervous system: role in brain inflammation and neurodegenerative diseases. Brain Res Brain Res Rev.

[52] van Kralingen C, Kho DT, Costa J, Angel CE, Graham ES (2013). Exposure to inflammatory cytokines IL-1beta and TNFalpha induces compromise and death of astrocytes; implications for chronic neuroinflammation. PLoS One.

[53] Meeuwsen S, Persoon-Deen C, Bsibsi M, Ravid R, van Noort JM (2003). Cytokine, chemokine and growth factor gene profiling of cultured human astrocytes after exposure to proinflammatory stimuli. Glia.

[54] Lynch MA (2015). Neuroinflammatory changes negatively impact on LTP: a focus on IL-1beta. Brain Res.

[55] Halassa MM, Haydon PG (2010). Integrated brain circuits: astrocytic networks modulate neuronal activity and behavior. Annu Rev Physiol.

[56] Jo S, Yarishkin O, Hwang YJ, Chun YE, Park M, Woo DH, Bae JY, Kim T, Lee J, Chun H (2014). GABA from reactive astrocytes impairs memory in mouse models of Alzheimer’s disease. Nat Med.

[57] Brogden RN, Speight TM, Avery GS (1975). Minocycline: a review of its antibacterial and pharmacokinetic properties and therapeutic use. Drugs.

[58] Yong VW, Wells J, Giuliani F, Casha S, Power C, Metz LM (2004). The promise of minocycline in neurology. Lancet Neurol.

[59] Kim HS, Suh YH (2009). Minocycline and neurodegenerative diseases. Behav Brain Res.

[60] Gonzalez-Perez O, Gutierrez-Fernandez F, Lopez-Virgen V, Collas-Aguilar J, Quinones-Hinojosa A, Garcia-Verdugo JM (2012). Immunological regulation of neurogenic niches in the adult brain. Neuroscience.

[61] Caggiano AO, Kraig RP (1996). Eicosanoids and nitric oxide influence induction of reactive gliosis from spreading depression in microglia but not astrocytes. J Comp Neurol.

[62] Yoon SY, Patel D, Dougherty PM (2012). Minocycline blocks lipopolysaccharide induced hyperalgesia by suppression of microglia but not astrocytes. Neuroscience.

[63] Ravizza T, Noe F, Zardoni D, Vaghi V, Sifringer M, Vezzani A (2008). Interleukin converting enzyme inhibition impairs kindling epileptogenesis in rats by blocking astrocytic IL-1beta production. Neurobiol Dis.

[64] de Lanerolle NC, Lee TS, Spencer DD (2010). Astrocytes and epilepsy. Neurotherapeutics.

[65] Gonzalez-Perez O, Lopez-Virgen V, Quinones-Hinojosa A (2015). Astrocytes: everything but the glue. Neuroimmunol Neuroinflamm.

[66] Devinsky O, Vezzani A, Najjar S, De Lanerolle NC, Rogawski MA (2013). Glia and epilepsy: excitability and inflammation. Trends Neurosci.

[67] Vezzani A, Moneta D, Conti M, Richichi C, Ravizza T, De Luigi A, De Simoni MG, Sperk G, Andell-Jonsson S, Lundkvist J (2000). Powerful anticonvulsant action of IL-1 receptor antagonist on intracerebral injection and astrocytic overexpression in mice. Proc Natl Acad Sci U S A.

[68] Auvin S, Shin D, Mazarati A, Sankar R (2010). Inflammation induced by LPS enhances epileptogenesis in immature rat and may be partially reversed by IL1RA. Epilepsia.

